# Ectopic pituitary neuroendocrine tumor arising in the sphenoid sinus with an avid 18F-fluorodeoxyglucose uptake masquerading as malignancy: A case report

**DOI:** 10.1016/j.radcr.2023.05.063

**Published:** 2023-06-17

**Authors:** Yukiko Usui, Ryo Kurokawa, Takahiro Fukushima, Richi Fujita, Reina Hosoi, Emi Miyawaki, Michio Hayashi, Sadahiro Kishisita, Mariko Kurokawa, Osamu Abe, Haruyasu Yamada

**Affiliations:** a Department of Radiology, NTT Medical Center Tokyo, Shinagawa-ku, Tokyo, Japan; b Department of Radiology, The University of Tokyo, 7-3-1 Hongo, Bunkyo-ku, Tokyo, 113-8655, Japan; c Department of Diabetes and Endocrinology, NTT Medical Center Tokyo, Shinagawa-ku, Tokyo, Japan; d Department of Otorhinolaryngology-Head and Neck Surgery, NTT Medical Center Tokyo, Shinagawa-ku, Tokyo, Japan

**Keywords:** Ectopic PitNET, Ectopic pituitary adenoma, Sphenoid sinus, 18F-fluorodeoxyglucose uptake, 18F-FDG PET/CT

## Abstract

Ectopic pituitary neuroendocrine tumors (PitNETs) are uncommon conditions that develop outside of the sella turcica. The sphenoid sinus is the most common site for ectopic PitNET, followed by the suprasellar region, clivus, and cavernous sinus. PitNETs, regardless of whether inside or outside sella, may display avid 18F-fluorodeoxyglucose (FDG) uptake and masquerade as malignant tumors. Herein, we report a case of ectopic PitNET arising in the sphenoid sinus, which was found as an FDG-avid mass during cancer screening. On magnetic resonance imaging, the tumor showed heterogeneous and intermediate signal intensity areas on T1- and T2-weighted images with cystic components, which was consistent with PitNET. The localization and the presence of empty sella were suggestive of ectopic PitNET, and the diagnosis of ectopic PitNET (prolactinoma) was confirmed by endoscopic biopsy. Ectopic PitNET should be considered in a mass similar in nature to an orthogonal PitNET in areas near the sella turcica especially in patients with empty sella.

## Introduction

Pituitary neuroendocrine tumors (PitNETs), formerly known as pituitary adenomas, are benign tumors that grow from pituitary gland cells and can cause various hormonal abnormalities. Ectopic PitNETs are uncommon conditions in which PitNETs develop outside of the sella turcica [1]. Ectopic PitNETs can occur in various sites, including the sphenoid sinus, suprasellar area, clivus, and cavernous sinus [2,3]. It is believed that these ectopic tumors arise from the aberrant migration of pituitary cells during embryonic development or the transformation of differentiated cells in ectopic sites [2,3]. Preoperative diagnosis of ectopic PitNETs is based on imaging features on magnetic resonance imaging (MRI) and computed tomography (CT), and hormonal examinations may be required to evaluate the tumor's hormonal activity [2,3]. Previous reports on imaging findings of ectopic PitNETs on 18F-FDG positron emission tomography (PET)/CT have been limited. Herein, we report a valuable case of ectopic PitNET initially found as 18F-fluorodeoxyglucose (FDG)-avid sphenoid mass on 18F-FDG PET/CT.

## Case presentation

**Fig. 1:**
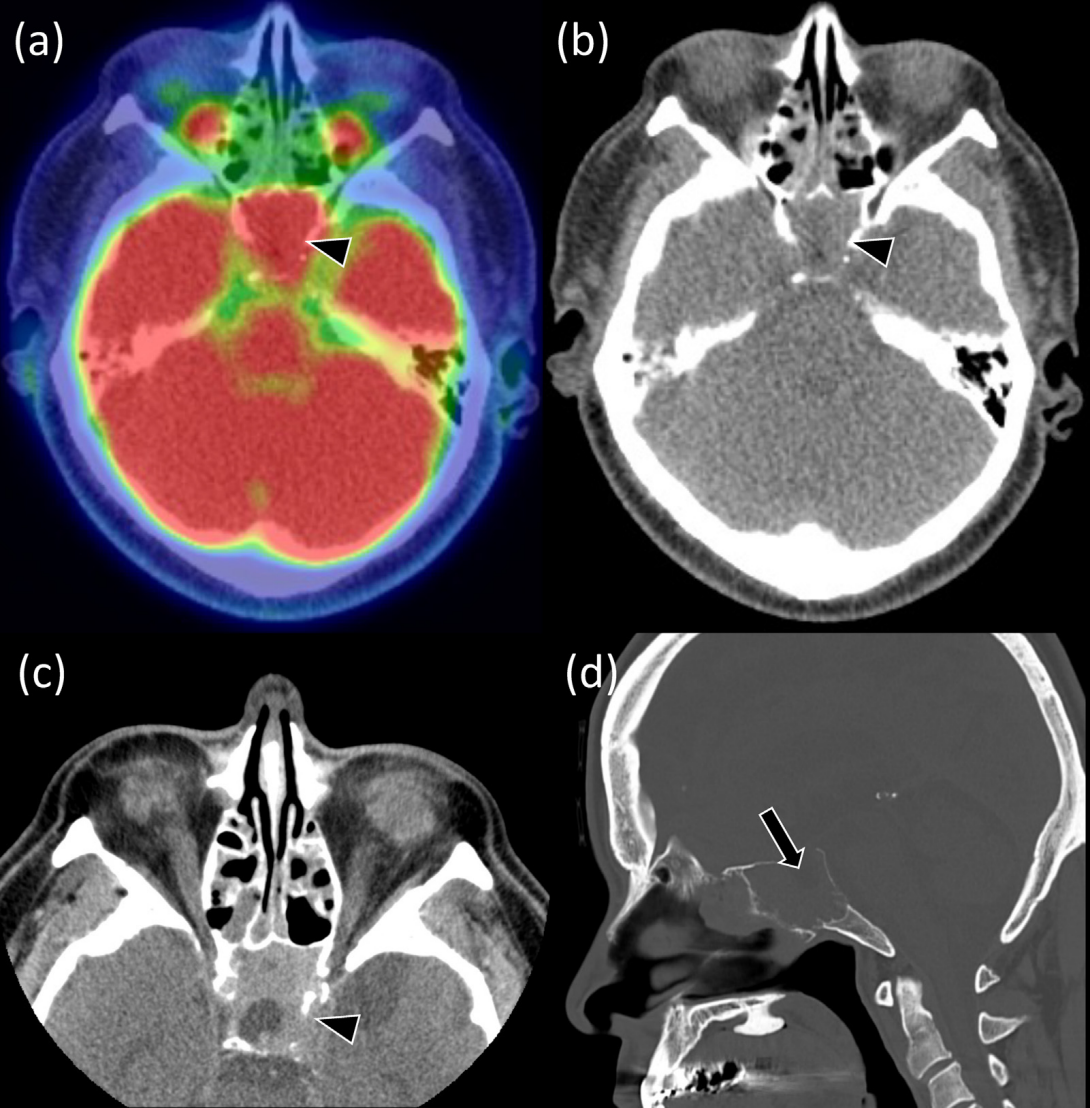
The mass in the sphenoid sinus on PET-CT and CT. 18F-FDG PET/CT shows soft density mass with a high FDG accumulation (SUV max, 16.1) filling the sphenoid sinus (A, B, arrowheads). The mass shows heterogeneous contrast enhancement on postcontrast CT (C, arrowhead). There are lytic changes in the clivus with a sclerotic rim, and the bone wall at the base of the sella turcica is hardly identified (D, arrow).

**Fig. 2:**
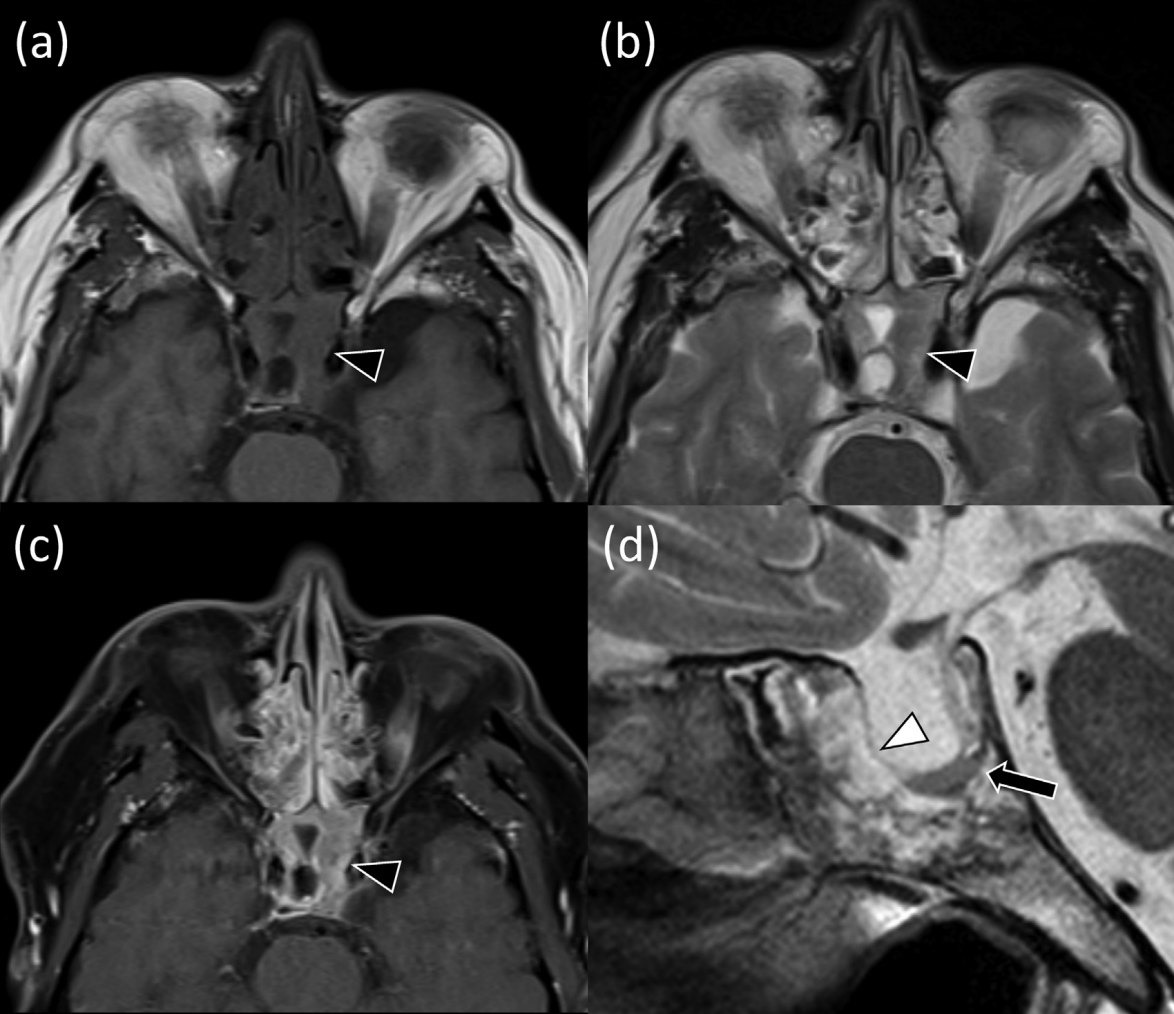
The mass in the sphenoid sinus on MRI. There is a mass in the sphenoid sinus with heterogeneous and intermediate signal intensity on T1-and T2-weighted images extending in the bilateral cavernous sinus (A, B, black arrowheads). Postcontrast T1-weighted image shows heterogeneous enhancement in the solid components and cystic components (C, black arrowhead). Sagittal T2-weighted image shows a thin anterior pituitary lobe in the basal portion of the enlarged sella turcica (D, arrow). T1-weighted hyperintensity area corresponding to the normal posterior pituitary lobe is absent (not shown). A thin T2-weighted hypointensity linear structure indicating a sella turcica dura mater is identified (D, white arrowhead).

A man in his 60s was referred to our hospital for further evaluation after a routine cancer screening revealed an abnormality in the sphenoid sinus. His past medical history included hypertension, atrial fibrillation (successfully treated with ablation at the age of 57 years), and vertigo, which had been investigated with an MRI at another facility, revealing only sinusitis. The 18F-FDG PET/CT examination performed during the cancer screening showed an FDG-avid mass in the sphenoid sinus (Fig. 1). The standardized uptake value (SUV) max was 16.1, which was higher than that of the brain parenchyma. The mass was heterogeneously enhanced and extended into the left cavernous sinus while thinning the base of the sella turcica. On MRI, the mass showed heterogeneous and intermediate signal intensity areas on T1- and T2-weighted images with contrast enhancement and cystic components without contrast enhancement (Fig. 2). A thin structure that appeared to be the anterior pituitary gland was identified at the base of the sella turcica, showing an empty sella. Normal T1-weighted hyperintense region in the posterior pituitary lobe was not observed in the vicinity of the tumor. A T2-weighted hypointensity linear structure suggesting the dura in the sella turcica was preserved, indicating that the mass was not an intrasellar tumor extending into the sphenoid sinus; rather, the mass was considered arising from the sphenoid sinus.

**Fig. 3:**
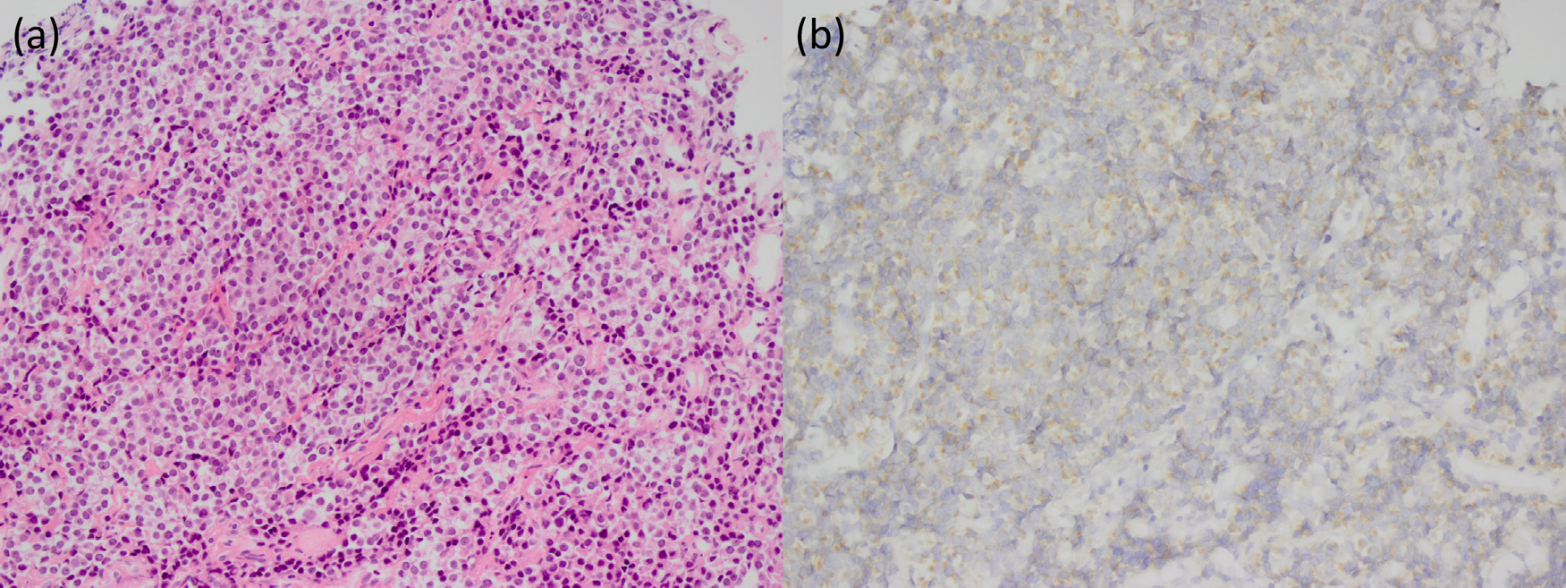
Pathology of the biopsied lesion. Hematoxylin and eosin staining shows an alveolar proliferation of atypical cells with hyperchromatic, enlarged, round-to-oval nuclei (A). These cells are positive for prolactin staining (B).

Endocrinological examination revealed abnormally high prolactin levels (2899.0 ng/mL; reference range: 3.6-12.8 ng/mL), without any related symptoms. Other hormone levels, including growth hormone, insulin-like growth factor I, adrenocorticotropic hormone, thyroid stimulating hormone, free T3, free T4, luteinizing hormone, and follicle-stimulating hormone, were within normal limits. An endoscopic sphenoidal biopsy was performed, and the tumor displayed an alveolar proliferation of atypical cells with hyperchromatic, enlarged round-to-oval nuclei (Fig. 3). The hyaline deposition was observed, but amyloid deposition was not confirmed using DFS staining. Immunohistochemical analyses revealed atypical cells positive for cytokeratin AE1/AE3, CD56, synaptophysin, chromogranin A (partial), and prolactin, while negative for CD3, CD20, and KP-1. The MIB-1 index was approximately 5%. Based on the radiological and pathological features, the final diagnosis of ectopic PitNET (prolactinoma) was made. Treatment with oral cabergoline (0.5 mg/wk) was initiated, and tumor size decreased and prolactin levels dropped to the normal level.

## Discussion

We report a case of ectopic PitNET (prolactinoma) arising in the sphenoid sinus initially detected on 18F-FDG-PET/CT as an FDG-avid mass.

PitNETs are benign tumors that grow from pituitary gland cells and can cause various hormonal abnormalities. PitNETs, formerly known as pituitary adenomas, are renamed in the fifth edition of the World Health Organization (WHO) classification 2021 [4,5] and 2022 World Health Organization Classification of Endocrine and Neuroendocrine Tumors [6,7]. The change in the nomenclature of this tumor was made based on the following clinical and pathological features of PitNETs inconsistent with “adenomas,” referring to benign epithelial tumors: PitNETs originate from neuroendocrine pituitary hormone-producing cells; some PitNETs can exhibit aggressive behaviors, such as infiltration into the surrounding structures, frequent recurrence after treatment, and occasional metastasis [8]. In addition, PitNETs express neuroendocrine proteins, such as synaptophysin, chromogranin A, CD56, and insulinoma-like protein 1, which are characteristics of neuroendocrine tumors [9,10].

The pituitary gland is composed of an anterior, a posterior, and an intermediate lobe. The adenohypophysis includes the anterior lobe, the pars intermedia, and the pars tuberalis.

The neurohypophysis includes the posterior lobe, the infundibular stalk, and the median eminence. Embryologically, the pituitary gland derives from the developing diencephalon (neurohypophysis) and the primitive oral stomodeum (adenohypophysis) [11]. Rathke's pouch originates from the primitive oral stomodeum and grows dorsally towards the CNS, separates from the stomodeum, and the 2 primordia approach and come into contact [11,12]. Ectopic PitNET is believed to originate from the embryonic Rathke's pouch and accounts for 0.48% (5/1024) of all PitNETs [13]. During the embryonic stage, a part of pituitary tissues can be retained in the sphenoid sinus or other pathways of Rathke's pouch, and PitNETs eventually develop [13]. The sphenoid sinus (34.4%) is the most common site for ectopic PitNET, followed by the suprasellar region (25.6%), clivus (15.6%), and cavernous sinus (13.3%) [2]. Other less common sites, such as the nasal cavity, superior orbital fissure, orbital cavity, maxillary sinus, and ethmoid sinus, have also been reported [2]. Epidemiologically, approximately 2:1 female-to-male predominance with the mean age at diagnosis of 45.4 years has been reported [2]. Approximately 60% of ectopic PitNETs are nonfunctional, but among functional tumors, adrenocorticotropic hormone (46%), followed by prolactin (26%) and growth hormone (22%), can be secreted [14].

On neuroimaging, ectopic PitNET shows no connection to the intrasellar pituitary gland. Nonenhanced CT typically shows a mass with isodensity compared to gray matter [3]. Bone involvement was more frequently observed in ectopic PitNETs arising in the clivus and sphenoid sinus than in ectopic PitNETs arising in other sites, and may invade the cavernous sinus or encase adjacent internal carotid arteries [2]. In the present case, ectopic PitNET arising in the sphenoid sinus progressed to the cavernous sinus. Ectopic PitNETs usually show intermediate or hypointensity on T1-weighted imaging and hyperintensity or intermediate intensity on T2-weighted imaging, with mild-to-moderate enhancement on postcontrast T1-weighted imaging as with the orthogonal PitNETs [2,3]. MRI tends to demonstrate multiple bubble-like or sometimes relatively large cystic foci of hyperintensity on T2-weighted imaging, which reflects histologically enlarged spaces filled with secretory granules [2]. The solid components and nonenhancing secretion-filled spaces make a cribriform appearance on MRI [2]. These MRI features were consistent with the present case. Ectopic PitNET in the sphenoid sinus (33.3%) and clivus (25.9%) tend to be with an empty sella, which was also observed in the present case [2]. The high frequency of empty sella may be due to the fact that most embryological cells remain in the sphenoidal or clival bone during migration [2]. Therefore, the empty sella may provide a diagnostic clue of ectopic PitNET.

The normal pituitary gland typically shows an FDG uptake of the background level on 18F-FDG PET/CT imaging [15]. A previous study reported that the most common causative disease of FDG uptake in the pituitary gland was a primary pituitary tumor, including PitNET (SUV max 13.6 ± 9.8), followed by metastatic malignancy (SUV max 16.0 ± 10.6), Langerhans cell histiocytosis (SUV max 15.0 ± 10.2), and hypophysitis [16]. Mean SUV max values tended to be higher in PitNETs larger than 10 mm (“macroadenomas”) than smaller or cystic lesions [17]. FDG uptake tended to be higher in nonfunctioning PitNETs than in functioning counterparts, without significant size difference between the two groups [17]. The information regarding SUV max of ectopic PitNET has been limited; however, Ishizaki et al. [18] reported an SUV max of 7.86 in ectopic PitNET arising in the sphenoid sinus.

## Conclusion

We reported a case of FDG-avid ectopic PitNET arising in the sphenoid sinus masquerading as malignancy. Ectopic PitNET should be considered in a mass similar in neuroimaging features to an orthogonal PitNET in areas near the sella turcica especially in patients with empty sella.

## Patient consent

The written informed consent was obtained from the patient for the publication of this case report and accompanying images.
